# The Basic Cardiovascular Responses to Postural Changes, Exercise, and Cold Pressor Test: Do They Vary in Accordance with the Dual Constitutional Types of Ayurveda?

**DOI:** 10.1155/2011/251850

**Published:** 2010-08-30

**Authors:** Piyush Kumar Tripathi, Kishor Patwardhan, Girish Singh

**Affiliations:** ^1^Department of Kriya Sharir, Faculty of Ayurveda, Institute of Medical Sciences, Banaras Hindu University, Varanasi, Uttar Pradesh, 221005, India; ^2^Division of Biostatistics, Department of Community Medicine, Institute of Medical Sciences, Banaras Hindu University, Varanasi, Uttar Pradesh, 221005, India

## Abstract

According to Ayurveda, the native Indian system of healthcare, three *Doshas*, namely, *Vata*, *Pitta*, and *Kapha*, are the basic mutually reciprocal mechanisms that are responsible for the maintenance of homeostasis in human beings. Ayurveda classifies entire human population into seven constitutional types *(Prakriti)*, based on the dominance of any single or a combination of two or three *Doshas*. Considering the fact that, in the recent past there have been several studies that have proposed some important genetic, biochemical and haematological bases for *Prakriti*, we conducted the present study in 90 randomly selected clinically healthy volunteers belonging to dual constitutional types (*Dvandvaja Prakriti*) to evaluate the variability of heart rate and arterial blood pressure in response to specific postural changes, exercise, and cold pressor test. The results of this study, in general, suggest that these basic cardiovascular responses do not vary significantly as per the dual constitutional types. However, we noted a significant fall in the diastolic blood pressure immediately after performing the isotonic exercise for five minutes, in *Vata-Kapha* individuals in comparison to the other two groups, namely, *Pitta-Kapha* and *Vata-Pitta*.

## 1. Introduction

According to Ayurveda, the native Indian system of medicine, the entire human population can be divided into seven constitutional types *(Prakriti) *[1–3]. As per this system, three mutually reciprocal mechanisms known as *Doshas, *namely, *Vata, Pitta, *and *Kapha *are responsible for the maintenance of homeostasis, and thus, the health [4]. Therefore, the constitutional types identified in Ayurveda are also based on the physiological dominance of any single or a combination of two or three* Doshas *in an individual [5]. Several mutually opposite attributes have been ascribed to these *Doshas *(Table 1), and each of these attributes produces a specific observable trait/feature/character (phenotype) in the individual. One's constitutional type is determined on the basis of these physical, physiological, and psychological features and this constitution, in turn, determines the predisposition to diseases, management, and the life-style regimen suitable for an individual [3]. The scheme of dividing the population into specific constitutional types is not unique to Ayurveda and is prevalent in other traditional systems of healthcare like Traditional Chinese Medicine, Kampo (the traditional medicine of Japan) and Sasang Constitutional Medicine of Korea as well [6–9]. The Hippocrates' model of four humours, the classification of human personality types by Kretschmer & Sheldon and the description of introversion–extroversion and neuroticism by Eysenck are all based on similar conceptual framework [10–13].

As per the explanations found in Ayurveda textbooks, the salient observable features among the individuals belonging to *Vata *constitution include: a weakly developed body build, irregular appetite, irregular food and bowel habits, rapid physical activities, dry skin and hair, and intolerance for cold temperature. The individuals belonging to *Pitta Prakriti *are characterised by the features like: high frequency and intensity of appetite and thirst, high tendency for perspiration, easy irritability, and good tolerance for cold temperature. On similar lines, *Kapha Prakriti *individuals are characterised by low appetite and digestion, less mobility, good memory, good resistance against diseases, and cool temperament [3]. 

In the recent past, there have been several interesting studies indicating either a genetic or a biochemical basis for these constitutional types described in Ayurveda. Udupa KN and others determined the basal blood levels of three neurotransmitters, namely, Acetylcholine, Catecholamines and Histamine along with the concerning enzymes like Cholinesterase, Monoamine oxidase and Histaminase and reported that the normal persons with features of *Vata, Pitta *and* Kapha* constitutions exhibited a relative preponderance of blood Cholinesterase, Monoamine oxidase and Histaminase activity, respectively [14]. Patwardhan B and others hypothesized that there could be a genetic basis for the three major constitutions described in Ayurveda. They evaluated 76 subjects both for their *Prakriti *and human leukocyte antigen (HLA) DRB1 types and observed a reasonable correlation between HLA type and *Prakriti *type [15, 16]. Prasher B and others observed that individuals from the three most contrasting constitutional types exhibited striking differences with respect to biochemical and hematological parameters and at genome wide expression levels. They also reported that biochemical profiles like liver function tests and lipid profiles and hematological parameters like hemoglobin levels exhibited differences between *Prakriti *types. Thus, they concluded that Ayurveda-based method of phenotypic classification of extreme constitutional types may be utilized to uncover genes that may contribute to system-level differences in normal individuals [3]. Ghodke Y and others carried out CYP2C19 genotyping in 132 unrelated healthy subjects of either gender, and, observed significant association between CYP2C19 genotype and major classes of *Prakriti* types. They reported that the extensive metabolizer genotype was found to be predominant in *Pitta Prakriti,* and the poor metabolizer genotype was highest in *Kapha Prakriti* when compared with other two *Prakriti* groups [17]. 

In spite of these reports, there are quite a few difficulties that have been encountered in assessing one's Ayurveda constitution. The accurate determination of one's constitution has been a problem in other traditional systems of medicine as well [18]. The age, physical and psychological status of the individual along with the season prevailing while assessing one's constitution are the major factors that tend to distort the outcome of this exercise. Differences in the subjective perceptions of the physicians also can make the assessment ambiguous. The absence of definite criteria for designating one's constitution to be either due to a single *Dosha* or due to two or three *Doshas *is another problem. Also, there is no data available to show the predominance of a particular type of *Prakriti *in a particular population. Nonavailability of the standardised and validated questionnaires to assess *Prakriti *makes the situation even more difficult. Most of the questionnaires available today are either based on too many textbooks or require physician-participant interaction in the form of a personal interview or even sometimes a detailed physical examination. This situation has resulted into the problems like: too lengthy questionnaires, inclusion of contradictory statements from different textbooks, and unnecessary divulgence of personal details of the participants. 

In the present study, we administered a modified version of the “Self-assessment questionnaire” [19] prepared originally by Patwardhan K and Sharma R to 150 randomly chosen healthy volunteers and shortlisted 90 volunteers who fulfilled the criteria to be designated as belonging to a dual type of constitution *(Dvandvaja Prakriti)*. We investigated for a possible correlation between these dual constitutional types and certain basic cardiovascular responses to postural changes, isotonic exercise (involving the shortening of muscles with the tone remaining constant), isometric exercise (involving the contraction of muscles without shortening), and cold pressor test. 

## 2. Objectives of the Study

Almost all the studies on *Prakriti* published so far have concentrated mainly on the individuals belonging to three extreme constitutional types, that is, *Vata, Pitta and Kapha *[3, 14–17]. In fact, as per our experience, the chances of encountering an individual with a truly extreme constitutional type are very rare. Though at variable degrees, one or the other secondary *Dosha *usually expresses its corresponding features in most of the individuals along with the primary *Dosha*. Therefore, we decided to design a study, where, only those individuals belonging to a dual constitutional type would be included. Further and more, no study available has so far concentrated on simple and clinically useful noninvasive parameters like heart rate (HR) and arterial blood pressure (ABP) variability in response to posture, exercise and cold pressor test. We hypothesised that these parameters, if showed some variability in accordance with the constitutional types, would serve as good indicators to determine and verify one's *Prakriti* along with offering some help in determining ones' predisposition to diseases. When parameters like blood levels of catecholamines show variability in accordance to *Prakriti, *it was logical to assume that some other basic clinical parameters might as well show similar variability.

With these objectives we planned the present study, where, 90 randomly selected clinically healthy volunteers belonging to the dual constitutional types were studied for the variability of HR and ABP in response to posture, exercise and cold pressor test.

## 3. Material and Methods

### 3.1. Population

Population for the present study was defined in terms of the students of either gender aged between 18–35 years and registered during the years 2008 and 2009 under the Bachelor of Ayurveda Medicine and Surgery (BAMS) and Ayurveda Vachaspati -MD(Ay)/ Ayurveda Dhanvantari-MS(Ay) programmes in the Faculty of Ayurveda, Institute of medical Sciences, Banaras Hindu University.

### 3.2. Randomization and Sampling Procedure

A list of students registered under BAMS and MD(Ay)/MS(Ay) courses was prepared, and all the names were entered in a column in the Microsoft Excel 2007 workbook. Then, the formula = RAND() was entered into the first cell of an empty column next to the column that was to be randomized. The RAND() function inserts a random number between zero and one into the cell. The whole column was then pasted with the RAND() function, and thus, a column of random numbers was generated. The entire sheet was then selected, and the data was sorted by the column containing the random numbers. Now, the complete list of the students stood randomized. The first 150 students from this list were administered with the self-assessment questionnaire to assess *Prakriti *after obtaining their written consent to participate in the study. This population included those freshly registered students who had no idea regarding the *Prakriti *(*n* = 55) and also those who had studied *Prakriti* thoroughly in their course of studies (*n* = 95). The clearance was obtained from the ethical committee of the University before the study was started.

### 3.3. Assessment of *Prakriti* among the Volunteers

#### 3.3.1. Preparation of the Questionnaire

We modified the “Self-assessment questionnaire for determining *Prakriti” *originally designed by Patwardhan and Sharma [19] and used it for the assessment of *Prakriti* of the individuals in the present study. This questionnaire is designed on the basis of the explanation found in *Charaka Samhita*, one of the most authentic textbooks on Ayurveda, authored around 1000 years BC [20]. This explanation is, in turn, based on the specific attribute *(Guna)* of a particular *Dosha* [5]*. Charaka Samhita *explains the specific attributes of a particular *Dosha *along with the description of the specific features these attributes produce in an individual. Thus, *Vata, Pitta,* and *Kapha* have been assigned with eight, five and twelve attributes *(Guna), *respectively. Furthermore, each attribute produces one or more observable features at the physical, physiological, or psychological domains.

Thus, three *Doshas *are neither assigned with equal number of attributes nor do they produce equal number of observable features in a given individual. This situation compelled us to adopt the self-assessment questionnaire which expresses the dominance of each *Dosha* in terms of percentage scores, and thus, makes the comparison of *Dosha *dominance quantitatively possible.

This questionnaire is designed in such a way that, each feature as described in *Charaka Samhita (Vimana Sthana) *has been converted into a simple question/statement. The respondents were required to record their agreement or disagreement with the statement/question in a column provided for the purpose in the form of “yes” or “no.” The scores to be allotted for a particular type of response were specified against the statement in a separate column. It was clearly mentioned that if the response of the individual was not the one mentioned in the column, the score to be allotted was zero.

We modified the said questionnaire to suit the needs of our study (see data-1 in Supplementary Material available online at doi: 10.1155/2011/251850). For the sake of convenience in the calculations, we allotted a total of 120 scores for each *Guna*. If a particular *Guna* produced a single feature, a full of 120 scores were allotted to that feature, when found in the individual. On the other hand, if a *Guna* produced more than one feature, 120 scores were divided equally into the specific number of features that the particular *Guna* produced. For example, if a particular *Guna* produced four features, 120 scores were divided by four, resulting in 30 scores for each feature. This new scoring pattern avoided the need of scores being allotted in fractional numbers. We also modified certain questions/statements to suit the mindset of the student population. Thus, the final maximal scores according to this questionnaire were 960 for *Vata*, 600 for *Pitta, *and 1440 for *Kapha*. The percentage dominance of a *Dosha* in an individual was calculated on the basis of the total scores obtained for each *Dosha* by simple mathematical calculation as shown in:

(1) $$\frac{\text{Total}\;\text{scores}\;\text{scored}\;\text{by}\;\text{an}\;\text{individual}\;\text{for}\;\text{a}\;Dosha\times 100}{\text{Total}\;\text{scores}\;\text{allotted}\;\text{to}\;\text{that}\;Dosha}.$$

At the end of this exercise, the respondents could calculate the scores for different *Doshas *by themselves and could understand the *Dosha* dominance in terms of percentage scores. As the questionnaire was of “Self-assessment” type, the respondents were not asked to submit their completed questionnaires; instead, they were asked to record and submit the *Dosha* predominance in the form of percentage scores. The advantage of doing so was that the respondents could answer all the questions honestly without having to face any questions from the interviewer. Also, this helped them to overcome any apprehension related to unnecessary divulgence of their personal details.

#### 3.3.2. Validation of the Questionnaire

To verify the results of this newly designed questionnaire, the questionnaire was correlated with another questionnaire that was already in use in the department of Kriya Sharir. The questionnaire that was already in use, required the physician-participant interaction and incorporated all the views expressed in different textbooks of Ayurveda. As this questionnaire too, gave the results in terms of percentage dominance of *Doshas, *we decided to administer both the questionnaires to 50 volunteers randomly. After this, the percentage scores recorded for *Vata, Pitta, *and *Kapha* were correlated using the software Statistical Package for Social Sciences (SPSS version 11.5), and the Pearson's Correlation Coefficient (*r*) was determined. At the end of this exercise, in all the three cases of *Vata, Pitta *and *Kapha*, we noted a positive correlation, and this correlation was significant as far as the measurement of *Vata *(*r* = 0.305, *P* = .015) and *Kapha *(*r* = 0.341, *P* = .031) was concerned. However, the correlation was not significant for *Pitta,* though the Pearson's Correlation Coefficient was positive (*r* = 0.237, *P* = .097). 

Validation of this kind of questionnaires remains to be a major problem in complementary and alternative medicine (CAM)- related research studies. The question as to whether the original questionnaire (with which the newly developed questionnaire was compared) was really accurate—remains unanswered in most of the cases. This is mostly because all such questionnaires try to measure some subjective parameters, and therefore, it is quite possible that the newly developed questionnaire is more sensitive than the original one, and yet, the correlation may still remain weak. Moreover, a correlation coefficient of 0.30 is usually taken as medium effect size in power analysis and sample size determination [21, 22]. On these considerations, we treated the newly designed questionnaire to be validated.

#### 3.3.3. Procedure Adopted to Assign the Dual Constitutional Type (*Dvandvaja Prakriti*) Status

The maximal (primary), moderate (secondary), and minimal (tertiary) *Doshas *were noted in terms of percentage scores and the following conditions were applied for designating an individual to be belonging to *Dvandvaja Prakriti*.

- The scores for the secondary Dosha should be at least 50% of the primary Dosha.
- The difference between the tertiary Dosha and the secondary Dosha should be at least 25% of the secondary Dosha.

Out of 150 volunteers, 90 individuals fulfilled both the above criteria and the *Prakriti *of these volunteers was designated as *Vata-Pitta, Pitta-Kapha, *or* Vata-Kapha*. While designating the *Prakriti, *the individuals with *Vata *as the primary *Dosha *and *Pitta* as the secondary *Dosha *(i.e., *Vata-Pitta* individuals, *n* = 4) and the individuals with *Pitta *as the primary *Dosha *and *Vata *as the secondary *Dosha *(i.e., *Pitta-Vata individuals, n* = 15) were considered to be equivalent and were grouped under *Vata-Pitta*. Similarly, the *Pitta-Kapha *individuals (*n* = 21) and the *Kapha-Pitta* individuals (*n* = 29) were treated to be equivalent and were grouped under *Pitta-Kapha. *On similar lines, the *Vata-Kapha *individuals (*n* = 5) and the *Kapha-Vata* individuals (*n* = 16) were treated to be equivalent and were grouped under *Vata-Kapha.* This was done to avoid the necessity of creating six groups of dual *Prakriti*, which would have been a deviation from the Ayurveda textbooks. 

These volunteers were enrolled in the study only after undergoing the clinical examination and being declared clinically healthy and physically fit. A written consent was obtained from them to participate in the study. These 90 volunteers were then subjected to some simple experiments in the human physiology laboratory of the department of Kriya Sharir as described in the following paragraphs.

### 3.4. Recording the Cardiovascular Responses

The volunteers were subjected to different tests in multiple sessions so that only one test was performed during one session. Each session was separated by a gap of at least 24 hours. The time of performing these tests was from four p.m. to five p.m. in the human physiology laboratory of the department of Kriya Sharir. All these tests were performed in the months of comfortable environmental temperature, that is, August, September, October, and November, thus, avoiding the extreme winters and summers.

#### 3.4.1. Recording the Basal Readings

Volunteers were asked to lie down in supine position for ten minutes and relax. After that, the arterial pulse rate per minute (PR), systolic blood pressure in mm Hg (SBP) and diastolic blood pressure in mm Hg (DBP) were recorded.

#### 3.4.2. Recording the Effect of Posture

- After recording the basal readings, the volunteers were asked to sit up quickly, and the PR, SBP, and the DBP were recorded immediately. After two minutes of assuming the sitting posture, the PR, SBP, and DBP were recorded once again.
- After this, the volunteers were asked to stand upright from the sitting posture as quickly as possible, and immediately, the PR, SBP, and DBP were recorded. The procedure of recording these parameters was repeated after two minutes of assuming the standing posture.
- After lying down in supine position for five minutes, the volunteers were asked to stand up quickly and the PR, SBP, and DBP were recorded as soon as possible. Procedure of recording these parameters was repeated after two minutes of standing upright.

#### 3.4.3. Recording the Effect of Isometric Exercise

- After five minutes of rest, the PR, SBP, and DBP of the volunteers were recorded in the standing upright position.
- The volunteers were asked to push the wall with both their hands as forcefully as possible for three minutes, that is, to perform isometric exercise.
- Immediately after the completion of exercise for three minutes, PR, SBP, and DBP of the volunteers were recorded.
- Procedure of recording the PR, SBP, and DBP was repeated after two and five minutes of completion of the isometric exercise.

#### 3.4.4. Recording the Effect of Isotonic Exercise

**Harvard Step Test**

In this test, the volunteers were asked to step up and down a 20-inch bench, at a frequency of 30 times/minute for five minutes [23]. If the subject felt breathlessness and exhausted, the test was stopped. Immediately after completion of five minutes of Harvard Step Test, the PR, SBP, and DBP were recorded. The procedure of recording the PR, SBP, and DBP was repeated after two and five minutes of completion of Harvard Step Test.

#### 3.4.5. Recording the Cardiac Efficiency Index (CEI)

To calculate the cardiac efficiency index, PR was recorded at the following three instances after the completion of Harvard Step Test, and the readings were designated as (a), (b), and (c):

- PR in between 1 and 1.5 minutes,
- PR in between 2 and 2.5 minutes,
- PR in between 3 and 3.5 minutes.

The cardiac efficiency index was calculated with the help of the following formula [23]:

(2) $$\frac{\text{Duration}\;\text{of}\;\text{Isotonic}\;\text{Exercise}\;\left(\text{in}\;\text{seconds}\right)\times 100}{\text{a}+\text{b}+\text{c}}=\ldots \%.$$

#### 3.4.6. Recording the Cold Pressor Test

In this test, right hand of the volunteer was immersed in cold water (4°C) kept in a tumbler till the level of wrist, and PR, SBP, and DBP were recorded in the left hand every 30 seconds for 2 minutes [24].

### 3.5. Calculations and Statistical Analysis

The data entry was carried out using the software Statistical Package for Social Sciences (SPSS version 11.5). The means were calculated for all the recorded parameters with respect to each *Prakriti *group. For the purpose of intergroup comparison, One-way ANOVA was used.

## 4. Observations

Out of the 90 volunteers in the study, 61 were males and 29 were females. The maximum number of volunteers belonged to the age group of 18–26 years (*n* = 72). When distributed in terms of constitutional types, the maximum number of volunteers were found to be belonging to *Pitta-Kapha Prakriti *(*n* = 50), and remaining volunteers were found to be distributed almost equally among *Vata-Kapha *(*n* = 21) and *Vata-Pitta *(*n* = 19) *Prakriti *groups.

It was interesting to note that, out of 150 volunteers who were screened initially, there was no individual who scored a zero for any *Dosha. *There was no individual who scored equal scores for all the three categories of *Doshas *either. The minimum percentage scores scored for a *Dosha *were 5% for *Vata,* 5% for *Pitta* and 20% for *Kapha. *In the same manner, the maximum percentage scores scored for a *Dosha* were 59% for *Vata*, 89% for *Pitta* and 81% for *Kapha*. This means that the role of *Vata, *as a factor determining one's constitution, was minimal in this sample.

Tables 2, 3, 4, 5, and 6 reveal that no parameter showed any significant variation in accordance with the *Prakriti* groups. However, as Table 7 suggests, the DBP recorded immediately after performing the isotonic exercise for five minutes showed a definite variation (*P* = .004) according to the constitutional type. The significant pairs were VP (mean DBP 97.79 ± 5.159) versus VK (mean DBP 92.00 ± 7.211) and PK (mean DBP 97.12 ± 6.183) versus VK (mean DBP 92.00 ± 7.211). This means that the fall in DBP was significant in VK group in comparison to VP and PK groups.

## 5. Discussion

**Figure 1:**
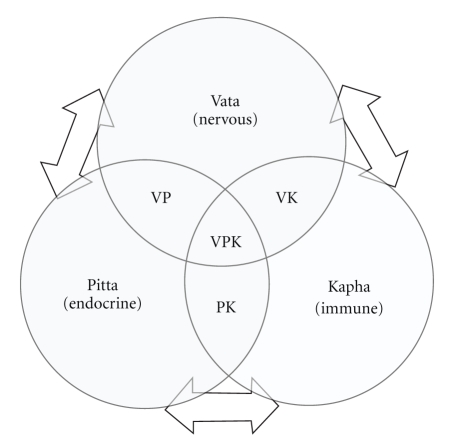


The entire description of human Physiology in Ayurveda is based primarily on the theory of *Tridosha*. The “homeostatic mechanisms” as conceptualised in modern biomedicine have a very close resemblance with this theory. On the basis of this understanding, the three *Doshas—Vata, Pitta *and* Kapha—*can be paralleled with the nervous, endocrine, and immune mechanisms, respectively [4]. This is because, *Vata *primarily initiates all the sensory and motor activities, *Pitta *controls digestion and metabolism, and *Kappha *confers immunity [4]. In fact, the modern biomedicine has only recently started taking note of the important interactions that take place between nervous, endocrine, and immune mechanisms and their role in the maintenance of homeostasis. For instance, the central nervous system influences the immune system through endocrine, paracrine and neuronal mechanisms. Immune cells produce a large number of endocrine hormones, neurotransmitters, and neuropeptides and are involved in the regulation of immunity. The endocrine and nervous systems express receptors for a wide variety of immunologically derived substances, suggesting potential regulatory feedback loops between these three major integrative bodily systems [4, 25]. Therefore, it can be presumed that the nervous, endocrine, and immune systems dominate the overall homeostatic regulatory scenario among the people with *Vata, Pitta *and *Kapha *types of constitutions respectively.  Figure 1 represents this hypothesis schematically. This understanding may support the view expressed by Hankey A that the CAM modalities can stimulate advances in theoretical biology [26].

Recently, there have been several efforts to provide an evidence base to the traditional systems of medicine [27–30], and a number of investigators have tried to provide some interesting genetic, biochemical, haematological or anatomical basis to the concept of Ayurveda constitution. But, most of the recent investigations carried out in relation to *Prakriti *have included only those individuals belonging to three extreme types of constitutions, which is comparatively a rare occurrence in our experience. Apart from this, the accurate assessment of one's constitution has always been a problem.

The present study tries to overcome most of the problems encountered while assessing one's *Prakriti *because the questionnaire used in this study is of “self-assessment” type and is based only on one classical textbook, that is, *Charaka Samhita*, and thus avoids the inclusion of contradictory views. Furthermore, the mathematical model incorporated in this questionnaire is based on the sound logic of “*Guna” *principle of Ayurveda. In this study, we included only those individuals with a dual type of constitution (produced due to the physiological dominance of two *Doshas*) and investigated for any possible correlation between the responses of certain basic cardiovascular parameters and the constitutional types.

Most of these basic cardiovascular responses that we studied have been proposed to have some predictive clinical significance. While Matthews and others suggest that the people who are at high risk for elevated blood pressure might have an exaggerated stress-induced cardiovascular response at a younger age [31], Sparrow and others advocate a possible relationship between the postural changes in diastolic blood pressure and the risk of subsequent myocardial infarction [32]. Ellestad and Wan, in 1975, showed that the lack of an appropriate heart rate response to exercise, termed “Chronotropic incompetence,” was associated with a greater risk of adverse cardiac events in the next five years than was ST segment depression [33]. Similarly, an exaggerated blood pressure response during exercise is probably an independent predictor of future hypertension [34, 35]. The clinical importance of the cold pressor test has been highlighted by Kasagi F and others. Their study supports the hypothesis that hyper reactivity to the cold pressor test is a predictor of the development of hypertension [36].

Though, the present study does not suggest any significant association between the *Prakriti *type and cardiovascular responses in the form of HR, SBP, and DBP to the changes in posture, isometric exercise, and cold pressor test, it suggests a possibility that the individuals with a dual type of constitution may differ significantly from those with an extreme type. It is being hypothesised that, the association of a secondary *Dosha *that has a nullifying/opposite effect due to the reciprocal relationship that it shares with the primary *Dosha, *may be responsible for this phenomenon. Therefore, it is being suggested that the prediction of any future morbidity based simply on *Prakriti *is difficult among the people belonging to the dual constitutional types. The study also suggests that the chances of encountering an individual with a truly extreme type of constitution are rare, and each *Dosha* probably contributes its share in framing ones' constitution, though at varying degrees.

A significant fall in DBP among *Vata-Kapha *individuals, recorded immediately after performing the isotonic exercise for five minutes, in comparison to *Pitta-Kapha* and *Vata-Pitta* individuals is another observation that is noteworthy. In fact, the isotonic exercise produces varying degrees of vasodilatation in different individuals. This is possibly related to the proportion of adrenaline and noradrenaline released into the blood stream during vigorous exercise. The affinities of these chemicals towards alpha and beta adrenergic receptors differ, and this may form the basis of the differences noted in the DBP response between different individuals [37]. As the study suggests, vasodilatation and resultant fall in peripheral resistance predominates among *Vata-Kapha *individuals after the isotonic exercise in comparison to the other two groups. This supports the Ayurveda theory that *Pitta *is related with aggressiveness because of its *Tikshna *(sharp) *Guna *[5]. An individual with *Pitta *dominant *Prakriti *is usually involved in risky/heroic acts and gets angry/irritated easily [5]. This observation may support the findings related to higher levels of catecholamines in *Pitta *individuals as suggested by Udupa KN and others [14]. As the role of *Pitta *in VK group is minimal in comparison to VP or PK groups, it may be presumed that the dominance of *Pitta *has got some positive relationship with adrenal medullary hormones, sympathetic activity and/or with such other mechanisms that regulate the total peripheral resistance. This factor may be taken as a lead and further studies may be designed to explore this relationship.

## 6. Summary and Conclusion

It may be concluded that the modified self-assessment questionnaire that we have used in the present study measures the dominance of *Dosha *to a fairly reliable extent and may be used to quantitatively express the *Dosha *dominance. Though, the study indicates that the basic cardiovascular responses do not significantly vary in accordance with the dual constitutional types of Ayurveda, it suggests that the fall in DBP recorded immediately after performing the isotonic exercise for five minutes varies significantly in relation to *Prakriti *groups and this fall is significant among VK group in comparison to VP and PK groups. This finding is indicative of some kind of positive association of *Pitta *with adrenal medullary hormones, sympathetic activity and/or such other mechanisms that regulate the total peripheral resistance. The study may also be considered as a lead to further investigations as to whether the individuals with a dual constitutional type differ significantly from those with an extreme constitutional type or not. A similar study, if carried out among the individuals belonging to truly extreme constitutional types, may throw some more light in this regard. 

## Supplementary Material

This is the modified version of “Self-assessment questionnaire to assess Prakriti” that we used in the present study. While using this questionnaire, the respondents are required to allot specific scores mentioned against each question/statement if their response is the one that is specified against each item. On the other hand, if the response is other than the one that is specified, the respondents are required to allot a score of zero. After completing the questionnaire, the respondents will be able to calculate the Dosha dominance using the formula that has been provided at the end.

## Figures and Tables

**Table 1:** The different attributes of three *Doshas* responsible for rendering them mutually reciprocal.

Vata	Pitta	Kapha
Nonunctuous/dry *(Ruksha) *	—	Unctuous *(Snigdha) *
Light *(Laghu) *	—	Heavy *(Guru) *
Cold *(Shita) *	Hot *(Ushna) *	Cold *(Shita) *
—	Pungent and sour* (Amla *and* Katu) *	Sweet *(Madhura) *
—	Liquid *(Drava) *	Solid *(Sandra) *
—	Sharp *(Tikshna) *	Dull *(Manda) *
Coarse/rough *(Parusha) *	—	Smooth *(Shlakshna) *
Nonslimy *(Vishada) *	—	Slimy *(Vijjala) *
Mobile *(Chala) *	—	Rigid *(Stimita) *
Abundant *(Bahu) *	—	—
Swift *(Shighra) *	—	—
**—**	Fleshy smell *(Visra) *	—
—	—	Essence *(Saara) *
—	—	Clear *(Accha) *
—	—	Soft *(Mridu) *

**Table 2:** The relationship between *Prakriti* and the basal readings of PR (per minute), SBP (mm Hg) and DBP (mm Hg).

Prakriti	PR (Mean ± SD)	SBP (Mean ± SD)	DBP (Mean ± SD)
VP (*n = *19)	79.42 ± 7.883	120.42 ± 9.535	75.47 ± 6.859
PK (*n = *50)	79.74 ± 8.444	116.52 ± 9.397	76.32 ± 7.153
VK (*n = *21)	78.76 ± 4.538	117.43 ± 9.384	72.67 ± 7.193
Intergroup comparison Oneway ANOVA	*P* = .885	*P* = .311	*P* = .146

**Table 3:** The effect of postural changes on PR (per minute), SBP (mm Hg), and DBP (mm Hg) in relation to *Prakriti*.

Posture change	Parameter	Time of recording	VP (*n = *19)	PK (*n = *50)	VK (*n = *21)	Intergroup comparison One-way ANOVA
Sitting from supine	PR (Mean ± SD)	Immediate	77.68 ± 7.580	79.08 ± 7.690	78.57 ± 5.662	*P* = .774
		After 2 minutes	81.95 ± 7.059	82.36 ± 8.218	82.57 ± 5.105	*P* = .963
	SBP (Mean ± SD)	Immediate	115.58 ± 9.651	111.12 ± 9.277	113.43 ± 9.320	*P* = .195
		After 2 minutes	122.32 ± 9.220	118.84 ± 9.373	121.14 ± 9.264	*P* = .329
	DBP (Mean ± SD)	Immediate	72.74 ± 6.674	73.24 ± 6.950	71.33 ± 6.950	*P* = .566
		After 2 minutes	76.32 ± 7.521	76.88 ± 6.517	75.81 ± 6.258	*P* = .819

Standing from sitting	PR (Mean ± SD)	Immediate	79.63 ± 7.159	79.98 ± 4.883	79.24 ± 4.300	*P* = .919
		After 2 minutes	82.00 ± 7.483	82.94 ± 8.102	82.86 ± 4.881	*P* = .890
	SBP (Mean ± SD)	Immediate	117.58 ± 10.167	113.48 ± 8.541	116.10 ± 10.010	*P* = .216
		After 2 minutes	123.05 ± 9.222	118.84 ± 8.563	121.62 ± 9.135	*P* = .166
	DBP (Mean ± SD)	Immediate	73.68 ± 6.156	74.64 ± 6.471	73.14 ± 6.343	*P* = .635
		After 2 minutes	76.21 ± 5.922	76.88 ± 6.223	75.33 ± 5.416	*P* = .606

Standing from supine	PR (Mean ± SD)	Immediate	78.32 ± 7.952	79.04 ± 7.848	79.81 ± 4.729	*P* = .810
		After 2 minutes	83.26 ± 7.279	83.03 ± 7.709	83.33 ± 4.520	*P* = .984
	SBP (Mean ± SD)	Immediate	115.58 ± 9.057	111.60 ± 8.843	116.29 ± 10.359	*P* = .089
		After 2 minutes	124.42 ± 9.179	119.64 ± 8.473	123.52 ± 9.735	*P* = .077
	DBP (Mean ± SD)	Immediate	72.53 ± 5.806	73.00 ± 6.315	72.48 ± 5.437	*P* = .926
		After 2 minutes	76.84 ± 5.937	76.68 ± 5.906	76.67 ± 4.830	*P* = .994

**Table 4:** The relationship between *Prakriti* and PR (per minute), SBP (mm Hg), and DBP (mm Hg) recorded at different time intervals after performing the isometric exercise for 3 minutes.

		VP (*n = *19)	PK (*n = *50)	VK (*n = *21)	Intergroup comparison One-way ANOVA
PR (Mean ± SD)	Immediate	88.74 ± 8.143	90.34 ± 8.070	89.14 ± 5.534	*P* = .680
	After 2 minutes	83.79 ± 8.080	84.80 ± 7.762	84.67 ± 5.598	*P* = .877
	After 5 minutes	80.53 ± 8.051	80.94 ± 7.133	81.52 ± 4.729	*P* = .898

SBP (Mean ± SD)	Immediate	131.47 ± 10.538	128.28 ± 10.222	130.38 ± 7.965	*P* = .430
	After 2 minutes	124.53 ± 9.137	121.44 ± 9.442	124.57 ± 8.322	*P* = .281
	After 5 minutes	120.63 ± 8.617	118.02 ± 8.904	122.10 ± 8.233	*P* = .168

DBP (Mean ± SD)	Immediate	90.00 ± 6.254	88.80 ± 7.825	88.10 ± 6.340	*P* = .700
	After 2 minutes	82.74 ± 6.118	81.48 ± 7.192	80.86 ± 7.227	*P* = .687
	After 5 minutes	80.00 ± 5.249	77.56 ± 8.450	77.71 ± 6.581	*P* = .465

**Table 5:** The relationship between *Prakriti* groups and cardiac efficiency index (CEI).

Prakriti	PR (per minute) recorded at different time intervals after			CEI (in %)
	completing the Harvard step test for 5 minutes			
	PR between 1–1.5 minutes (Mean ± SD)	PR between 2–2.5 minutes (Mean ± SD)	PR between 3–3.5 minutes (Mean ± SD)	Cardiac efficiency index (Mean ± SD)
VP (*n = *19)	98.00 ± 6.110	92.42 ± 6.203	88.47 ± 6.168	107.77 ± 7.425
PK (*n = *50)	100.06 ± 8.052	93.36 ± 7.105	87.36 ± 3.766	106.50 ± 8.443
VK (*n = *21)	98.81 ± 6.493	93.14 ± 5.121	89.05 ± 4.318	106.84 ± 5.672
Intergroup comparison One-way ANOVA	*P* = .545	*P* = .867	*P* = .818	*P* = .830

**Table 6:** The relationship between *Prakriti* and PR (per minute), SBP (mm Hg), and DBP (mm Hg) recorded at different time intervals during the cold pressor test.

Parameter	Time of recording	VP (*n = *19)	PK (*n = *50)	VK (*n = *21)	Intergroup comparison One-way ANOVA
PR (Mean ± SD)	After 30 seconds	82.21 ± 7.598	83.20 ± 7.751	83.24 ± 4.358	*P* = .861
	After 60 seconds	89.16 ± 7.669	89.96 ± 7.956	89.62 ± 4.318	*P* = .917
	After 90 seconds	94.68 ± 6.717	95.48 ± 7.841	94.29 ± 4.660	*P* = .783
	After 120 seconds	95.53 ± 6.915	95.40 ± 7.892	94.10 ± 4.625	*P* = .748

SBP (Mean ± SD)	After 30 seconds	125.47 ± 8.161	121.24 ± 8.989	123.71 ± 8.131	*P* = .165
	After 60 seconds	132.11 ± 9.273	128.68 ± 9.576	130.48 ± 8.195	*P* = .365
	After 90 seconds	138.11 ± 8.232	134.16 ± 9.213	134.86 ± 7.472	*P* = .240
	After 120 seconds	138.42 ± 8.044	134.12 ± 9.358	135.71 ± 7.135	*P* = .183

DBP (Mean ± SD)	After 30 seconds	79.26 ± 7.187	78.84 ± 6.810	76.10 ± 6.276	*P* = .237
	After 60 seconds	83.05 ± 6.745	83.00 ± 6.812	80.19 ± 6.129	*P* = .239
	After 90 seconds	85.89 ± 6.879	84.88 ± 6.942	82.86 ± 6.650	*P* = .352
	After 120 seconds	86.95 ± 6.811	85.28 ± 6.758	82.67 ± 6.272	*P* = .123

**Table 7:** The relationship between *Prakriti* and PR (per minute), SBP (mm Hg) and DBP (mm Hg) recorded at different time intervals after performing the isotonic exercise for 5 minutes.

		VP (*n = *19)	PK (*n = *50)	VK (*n = *21)	Intergroup comparison	

					One-way ANOVA	Post hoc test (LSD) significant pairs (*P* < .05)
PR (Mean ± SD)	Immediate	103.16 ± 5.824	104.84 ± 9.303	104.29 ± 8.632	*P* = .765	—
	After 2 minutes	92.79 ± 6.469	93.94 ± 7.490	94.19 ± 5.862	*P* = .785	—
	After 5 minutes	82.95 ± 5.512	84.12 ± 6.483	84.86 ± 4.408	*P* = .586	—

SBP (Mean ± SD)	Immediate	148.84 ± 6.677	147.24 ± 8.627	143.05 ± 7.473	*P* = .056	—
	After 2 minutes	136.21 ± 6.494	134.92 ± 8.864	132.29 ± 7.932	*P* = .295	—
	After 5 minutes	125.26 ± 6.402	123.84 ± 8.210	123.43 ± 6.266	*P* = .709	—

DBP (Mean ± SD)	Immediate	97.79 ± 5.159	97.12 ± 6.183	92.00 ± 7.211	***P* = .004**	VP versus VK and PK versus VK
	After 2 minutes	88.63 ± 4.810	88.80 ± 5.628	85.43 ± 6.547	*P* = .070	—
	After 5 minutes	81.68 ± 4.773	80.84 ± 5.270	78.38 ± 6.249	*P* = .122	—
