# Supplementary material for: The Basic Cardiovascular Responses to Postural Changes, Exercise, and Cold Pressor Test: Do They Vary in Accordance with the Dual Constitutional Types of Ayurveda?
Source: Evid Based Complement Alternat Med. 2010 Aug 30;2011:251850. doi: 10.1155/2011/251850 (PMC2952295; doi:10.1155/2011/251850)
Supplement: Supplementary file 1 — This is the modified version of “Self-assessment questionnaire to assess Prakriti” that we used in the present study. While using this questionnaire, the respondents are required to allot specific scores mentioned against each question/statement if their response is the one that is specified against each item. On the other hand, if the response is other than the one that is specified, the respondents are required to allot a score of zero. After completing the questionnaire, the respondents will be able to calculate the Dosha dominance using the formula that has been provided at the end. [file 251850.f1.pdf]

The self-assessment questionnaire to assess *Prakriti**

Sl. No.	Question/statement	*Guna*	Is your answer the following?	Scores to be allotted if your answer is the one that is mentioned in the previous column	Allot the Scores in this column
		KAPHA			
1.	Whether your skin remains oily throughout the year in comparison to others?	*Snigdha*	Yes	120	
2.	Are your body-hairs & skin shiny, even when no oil or moisturizer is used?	*Shlakshna*	Yes	120	
3.A.	Are you considered attractive among your friends?	*Mridu*	Yes	40	
3.B.	Do even mild or trivial injuries on your body make you upset?	*Mridu*	Yes	40	
3.C.	Among your family members, is your complexion considered fairer?	*Mridu*	Yes	40	
4.	Do you think you have intense sexual desire?	*Madhura*	Yes	120	
5.A.	Have you got well built muscles?	*Saara*	Yes	60	
5.B.	Do you change your body posture frequently? (You cannot manage yourself in a stable posture for a long duration.)	*Saara*	No	60	
6.	Do you have a well-nourished & normally developed body? (You are neither malnourished nor obese.)	*Sandra*	Yes	120	
7.A.	Are you lazy and disinterested in activities like morning walk/ jogging, swimming or any type of outdoor games?	*Manda*	Yes	40	
7.B.	Are you slow in consuming the food? (Even after all have left the dining hall, you are still consuming the same amount of food).	*Manda*	Yes	40	

7.C.	When you go to morning walk or college or office, do you walk slowly in comparison to others?	*Manda*	Yes	40	
8.A.	If you are assigned any work, do you take some extra time to start it?	*Stimita*	Yes	40	
8.B.	Do you get irritated easily? (For example, when you don't get breakfast on time in your hostel or when the power goes off while watching a cricket match or your favourite movie on television).	*Stimita*	No	40	
8.C.	Are you late to develop/suffer from symptoms after exposure to common causative factors? (For example, during seasonal changes, when your friends are easily caught up with flu etc., you are still healthy among them).	*Stimita*	Yes	40	
9.	Does your gait (style of walking) change with respect to speed or manner frequently?	*Guru*	No	120	
10.A.	Do you feel hungry more frequently and do you consume more food in comparison to others?	*Shita*	No	30	
10.B.	Do you tolerate heat easily?	*Shita*	Yes	30	
10.C.	Do you consume liquids in more quantity and frequency in comparison to others?	*Shita*	No	30	
10.D.	Do you perspire less in comparison to others?	*Shita*	Yes	30	
11.	Are sounds produced frequently in your joints on movement?	*Picchila*	No	120	
12.A.	Have you got a good/ attractive complexion?	*Achcha*	Yes	60	
12.B.	Have you got sweet & pleasant voice?	*Achcha*	Yes	60	
				**1440**	**Total=**
PITTA					
1.A.	Are you more comfortable in winter than summer?	*Ushna*	Yes	17	

1.B.	Among your family members, is your complexion considered fairer?	*Ushna*	Yes	17	
1.C.	Does your temperature of oral cavity remain towards upper limit of normal range?	*Ushna*	Yes	17	
1.D.	Do you have excessive black moles, Freckles etc on your skin?<br>Or<br>Have you noticed new appearance of black moles often on your skin?	*Ushna*	Yes	17	
1.E.	Do you feel excessive hunger & thirst in comparison to others?	*Ushna*	Yes	17	
1.F.	Have you experienced premature graying, wrinkling of skin & early baldness?	*Ushna*	Yes	17	
1.G.	Do you have soft, scanty, brown hair on your face, body & head?	*Ushna*	Yes	17	
2.A.	Do you involve yourself in risky & heroic activities requiring physical strength often?	*Tikshna*	Yes	24	
2.B.	Do you have ability to digest large quantities of food easily?	*Tikshna*	Yes	24	
2.C.	Do you take large quantities of food & drink in comparison to others?	*Tikshna*	Yes	24	
2.D.	Do you get easily irritated for small/negligible problem in day-to-day life?	*Tikshna*	Yes	24	
2.E.	Do you consume food more frequently than others? (5-6 times/day)	*Tikshna*	Yes	24	
3.A.	Do you have soft & loose muscle bulk especially around the joints?	*Drava*	Yes	60	
3.B.	In comparison to others do you pass urine & stool in large quantities and do you perspire more?	*Drava*	Yes	60	

4.	Do your friends complain of bad smell being emitted from your body & mouth?	*Visra*	Yes	120	
5.	Do you think you have intense sexual desire?	*Amla+ Katu*	No	120	
				**600**	**Total=**
VATA					
1.A.	Whether your skin remains dry throughout the year in comparison to others?	*Ruksha*	Yes	30	
1.B.	Is your body undernourished/ emaciated?	*Ruksha*	Yes	30	
1.C.	Have you got rough, low, broken or obstructed voice?	*Ruksha*	Yes	30	
1.D.	Does Your sleep last less than 6 hours per day? Or Can your sleep be disturbed easily?	*Ruksha*	Yes	30	
2.A.	Do you change walking speed & style from time to time?	*Laghu*	Yes	40	
2.B.	Do you keep changing your food habits from time to time?	*Laghu*	Yes	40	
2.C.	Do you keep changingyour walking / jogging habit from time to time?	*Laghu*	Yes	40	
3.	Do you keep your joints, eyes, eyebrows, jaw, lips, tongue, head, Shoulder, hands & feet frequently moving?	*Chala*	Yes	120	
4.A.	Are you considered a talkative among your friends?	*Bahu*	Yes	60	
4.B.	Do you have prominent veins & tendons all over the body?	*Bahu*	Yes	60	
5.A.	Do you generally start the work assigned to you immediately?	*Shighra*	Yes	15	
5.B.	Do you get irritated easily? (E.g., when you do not get breakfast on time in your hostel or when the power goes off while watching a cricket match or your	*Shighra*	Yes	15	

	favourite movie over television)				
5.C.	Do you get frightened easily?	*Shighra*	Yes	15	
5.D.	Do you make friends easily & also lose them easily?	*Shighra*	Yes	15	
5.E.	Do you generally learn things quickly?<br>Or<br>Do you have a good grasping power?	*Shighra*	Yes	15	
5.F.	Is your long term memory weak? (E.g., you can remember only few names of your friends at your primary school).	*Shighra*	Yes	15	
5.G.	Are you more comfortable in summer?<br>Or<br>Do you prefer hot/warm drinks over cold drinks?	*Shighra*	Yes	15	
5.H.	Do you generally develop symptoms immediately after exposure to common causative factors?(You are easily caught by diseases like flu, allergy during seasonal changes).	*Shighra*	Yes	15	
6.A.	Do you shiver in winter season more than your friends?	*Shita*	Yes	60	
6.B.	Do you often feel stiffness in your body after exercise, travelling?	*Shita*	Yes	60	
7.	Are your nails, teeth, hands, feet and hairs on your body and face rough?	*Parusha*	Yes	120	
8.A.	Do you have cracks on the body especially on the heels?	*Vishada*	Yes	60	
8.B.	Are some crackling sounds produced in your joints during movements?	*Vishada*	Yes	60	
				**960**	**Total=**

$$\frac{Total\ scores\ scored\ by\ an\ individual\ for\ a\ Dosha}{Total\ scores\ allotted\ to\ that\ Dosha} x100$$

Final percentage scores for*Vata* =
Final percentage scores for*Pitta*=
Finalpercentage scores for*Kapha*=

*Originally prepared by: **Kishor Patwardhan** and **Rashmi Sharma.** Modified by: **Piyush Kumar Tripathi, Kishor Patwardhan** and **Girish Singh,** Institute of Medical Sciences, Banaras Hindu University, Varanasi.
